# The Impact Mechanism of Government Regulation on the Operation of Smart Health Senior Care Service Platform: A Perspective From Evolutionary Game Theory

**DOI:** 10.34172/ijhpm.8646

**Published:** 2025-02-22

**Authors:** Meng Xiao, Huan Liu

**Affiliations:** School of Management, Shenyang University of Technology, Shenyang, China.

**Keywords:** Smart Health, Senior Care Platform, Regulation, Opportunism, Evolutionary Game

## Abstract

**Background:**

Smart health and senior care services have developed rapidly to cater to the aging society, but the corresponding regulations are not perfect. The platform and senior care service enterprises have chosen different strategies due to various factors, resulting in disorderly platform development and inefficient resource allocation. This research attempts to establish a regulatory mechanism to promote the active development of the platform.

**Methods:**

In order to mitigate opportunistic behaviors in the smart health senior care service platform, drawing from evolutionary game theory, this study establishes a decision-making model involving the government, the platform, and senior care service enterprises, and scrutinizes the evolutionary behaviors and equilibrium strategies of these three parties.

**Results:**

There are five equilibrium points among the three parties in the game, and the strategy selection shows periodic changes. The cost and level of positive government regulation, the conversion rate of smart aging construction services provided by the platform, penalties for opportunistic behavior by the platform, as well as the benefits of positive cooperation and penalties for passive cooperation by senior care service enterprises, will significantly affect the strategies of all parties involved.

**Conclusion:**

The research results indicate that establishing a regulatory mechanism for the smart health senior care service platform can promote effective cooperation between platform and senior care service enterprises. Active government regulation is the key to the smooth operation of the platform. Through quantitative analysis, the main strategic choices of participants in the smart health senior care service platform can be explained. This model can provide a reference for formulating policies related to smart senior care.

## Background

###  The Current Development Status of the Smart Elderly Care Market

**Key Messages**

**Implications for policy makers**

- Effective government regulation is crucial for the platform’s smooth operation.
- Policies developed to regulate the development of the smart senior care market should consider how to mobilize the enthusiasm of both the platform and senior care service enterprises.
- The cost and level of positive government regulation, the conversion rate of smart senior care construction services of the platform, the rewards for reciprocity and the penalties for opportunism of the platform, and the benefit for positive cooperation and the punishment for negative cooperation of senior care service enterprises are important factors affecting the strategies of all parties involved.
- The establishment of a supervision mechanism for the smart health senior care service platform can promote effective cooperation between the platform and senior care service enterprises.

**Implications for the public**

This study may benefit the public through better government decision-making. By using data released from various studies, this study explains the changes in platform opportunism tendencies and the attitudes of senior care service enterprises towards cooperation. In this process of change, the ultimate cooperation and win-win situation between the platform and senior care service enterprises is also beneficial for the public to enjoy better quality smart senior care services. The public may use this study to scrutinize the behavior of all parties on the platform, thereby better avoiding opportunistic behavior.

According to the World Bank database, as of 2022, the proportion of individuals aged 65 and above has exceeded 9% of the total population. With the advancement of society, the requirement for health and senior care services among the populace is steadily increasing. However, the availability of societal resources falls short, leading to the prominent imbalance in the supply and demand in health and senior care services. To improve the efficiency of service resource utilization, continual enrichment of health and senior care products and services is imperative.

 Smart senior care refers to the utilization of modern information technology to establish close connections among the senior, government, communities, and medical institutions.^1^ The smart health senior care service platform integrates sensors, modern mobile devices, and communication technologies, facilitating senior individuals to receive medical monitoring and health services within the comfort of their homes.^2,3^ Presently, smart senior care has been preliminarily implemented in the lives of seniors, such as China’s pilot construction of smart towns in Guangxi, Sichuan, Yunnan, and other provinces.^4^ Brazil employs environmental assisted living to address the physical condition and home environment of the senior,^5^ while Sweden promotes e-home care utilizing information and communication technologies.^6^ From the perspective of demand, the smart health senior care service platform caters to the needs of the senior,^7^ healthcare,^8^ mutual assistance,^9^ and other necessities to improve the quality of life for seniors. From the perspective of operation, the platform can employ the online to offline model to connect the senior with service providers, effectively addressing the supply-demand imbalance.^10^ As of 2023, the total patent value of the global smart senior care industry is $42.055 billion.

 Due to the challenges posed by the large-scale construction, substantial investment requirements, and stringent technical prerequisites in establishing a smart health senior care service platform, as well as the absence of standardized criteria for participants involved in supplying smart senior care services, suppliers of smart services and products are still grappling with issues in production management, quality standards, after-sales evaluation, and ethical disputes.^11^ It proves challenging to achieve high-quality and high-level construction solely with limited financial investments and technical expertise from local governments. In the long term, the construction and operation of the smart health senior care service platform may encounter disorderly development, resulting in losses for all stakeholders involved. Therefore, there is the pressing need to delve deeply into strategies for actively developing the smart health senior care service platform under regulation.

###  Smart Health Senior Care 

 In contrast to the traditional pension model, the smart health senior care service platform transitions from fragmented to integrated supply of smart senior care services.^12^ While digital empowerment theoretically addresses some limitations of the traditional senior care model, smart senior care services primarily target the young and healthy senior population in practice.^13^ Some studies indicate that senior individuals generally possess limited proficiency in utilizing information tools compared to the middle-aged and younger counterparts.^14^ Furthermore, certain devices and programs often fail to meet the needs of senior individuals, particularly those who are disabled, terminally ill, or residing in remote areas.^15^ Additionally, some senior individuals harbor concerns regarding physical and mental safety risks, privacy breaches, deceptive consumption practices, and potential online fraud due to the privacy policies enforced unfavorably by senior care service enterprises.^16,17^

 The development of smart health senior care projects has emerged as a significant area of study in recent times. Some scholars advocate for the encouragement of increased stakeholder participation in the smart health senior care service platform through policy interventions and other measures.^18^ Moreover, appropriate social support behaviors, such as economic assistance and psychological encouragement, can assist senior individuals in overcoming barriers to utilizing information technology.^19^ However, research on smart senior care has mainly centered on the advancement of intelligent technologies and devices.^20^ Examples include the introduction of nursing robots aimed at enhancing the morale of nursing staff in senior care facilities,^21^ and the development of measurement tools designed to discern the preferences of the senior and provide them with a seamless experience.^22^

###  Regulatory of Senior Care Platform

 As a burgeoning sector within the platform economy, the construction of senior care platform faces numerous challenges stemming from market dynamics and institutional frameworks as smart health senior care service platform is increasingly promoted and utilized. Existing literature reflects the active engagement of the academic community in exploring the stakeholders and associated issues surrounding smart senior care. Scholars have developed the multi-resource party symbiotic evolution model for the smart senior care service paradigm based on symbiosis theory and logical growth models.^23^ Additionally, others have investigated the factors influencing user adoption of smart home medical services using multi-model theories such as the technology acceptance model, rational action theory, and planned behavior theory.^24^ Furthermore, some researchers have evaluated and analyzed the quality of smart community senior care services utilizing the fuzzy grey two-stage decision-making model.^25^ Although these methods provide reference for improving the smart elderly care system, most of them use static research to explore how to take measures to improve the quality of smart elderly care services, and focus on a single party perspective. The operation of smart senior care service platform is different from traditional senior care services, as it requires the participation of multiple parties. Therefore, when analyzing the regulation of smart health senior care service platform, the dynamic mutual influence of multiple party strategies should be considered. Evolutionary game theory can deduce the strategic evolution of each party in the smart health senior care service platform system, and more significantly demonstrate the role of government regulation in the smart health senior care service platform system. And some scholars have demonstrated the applicability of evolutionary game theory in the research of smart city regulation^26^ and vaccination arrangement management.^27^ Therefore, adopting evolutionary game theory to analyze the regulatory mechanism of smart health senior care service platform is a dynamic and multi-party extension of research methods in this field.

 The establishment and functioning of the smart health senior care service platform hinge on the trust of the elderly individuals to the government. Collaborating with enterprises, the government must adapt various regulations to oversee participant interactions and actively intervene when necessary. Implementing the smart senior care strategy should entail the government-led approach with multi-party involvement in artificial intelligence senior care governance.^28^ Effective cooperation mandates the adoption of process-oriented or outcome-focused regulatory measures by the government to safeguard the public welfare of smart senior care projects and prevent platform from excessively prioritizing maximum benefits at the expense of public interests. Introducing third-party regulations can mitigate government regulatory costs.^26^ However, the crux of ensuring the normal functioning of smart health senior care service platform lies in the collaboration between digital platform and smart health senior care service enterprises guided by the concept of value co-creation. Establishing standards for smart health senior care service platform and implementing the “reward for compensation” mechanism for platform operation are essential.^29^ Moreover, systematic strategy selection under government intervention is imperative to approach the desired state.^12^

 In summary, existing literature predominantly employs static research methods to explore avenues for enhancing the quality of smart health senior care services,^30^ with dynamic research being relatively scarce. While some studies endeavor to investigate the regulation of senior care service quality, they offer insights into the behavioral decision-making of participants in smart health senior care service platform. For instance, discussions on home care services have introduced third-party evaluation institutions to construct the third-party game model, underscoring the government’s inevitable regulatory responsibility.^31^ However, existing research primarily focuses on the behavior of individual participants within the traditional pension model, neglecting to address the strategies of each participant and their impact within the context of smart pension. In the era of rapid digital technology advancement, further exploration is required to decipher the strategic choices of participants in the operation of smart health senior care service platform, particularly their behavioral decisions under conditions of limited rationality.

###  Research Question of This Paper

 In light of the above, this study delves into the developmental landscape of smart senior care, focusing on the government, platform, and senior care service enterprises as the focal points of investigation, utilizing evolutionary game theory to examine the decision-making behaviors of the three parties. The innovation lies in two key aspects: (1) Evolutionary game theory combines game theory analysis with dynamic evolution process analysis, emphasizing dynamic equilibrium rather than the static equilibrium in traditional game theory.^32^ From the perspective of bounded rationality, employing evolutionary game theory to analyze the strategic choices and evolutionary behaviors among the government, platform, and senior care service enterprises. This exploration aims to identify feasible conditions for “positive government regulation, platform reciprocity, and senior care service provider positive cooperation,” thereby enriching the theoretical research on the operation and regulation of smart health senior care service platform. (2) Considering the impact of various factors such as government regulatory costs, the extent of platform aging construction, and the incentives and punishments for platform and senior care service enterprises, the revenue model for the government, platform, and senior care service enterprises is formulated. Through the analysis of multiple factors, the construction and application of the regulatory mechanism for the smart health senior care service platform are examined.

 This study aims to explore the regulatory mechanisms for the construction and operation of the smart health senior care service platform, seeking to answer three questions: (1) Under what circumstances are governments inclined to actively regulate the platform and senior care service enterprises? (2) How does the decision-making behavior of platform and senior care service enterprises evolve under positive government regulation? (3) What viable strategies exist to achieve positive government regulation, platform reciprocity, and cooperation from senior care service enterprises? By addressing these questions, this study aims to offer practical suggestions for establishing the regulatory mechanism for smart health senior care service platform, promoting the healthy growth of the silver economy, and fostering the conducive environment for the senior to enjoy their later years.

## Methods

###  Problem Description

**Figure 1:**
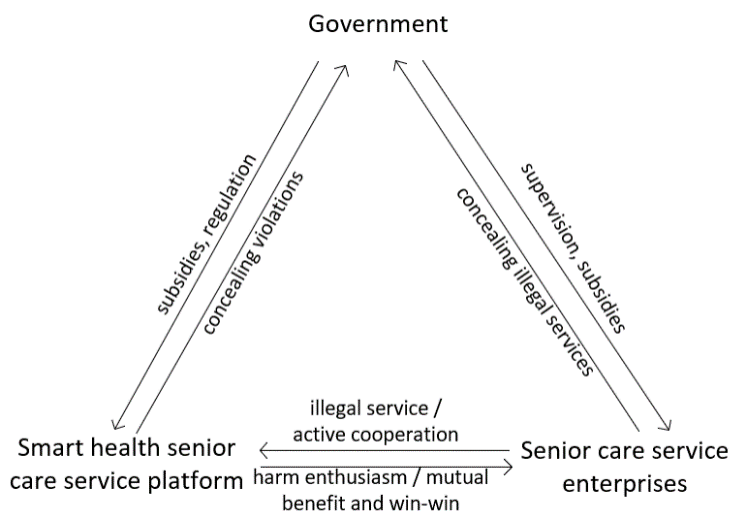
Logic Relationship of Game Model for Smart Health Senior Care Service Platform. Among them, the government delineates the responsibilities and operational standards for the establishment of the smart health senior care service platform. The platform utilizes technologies such as the Internet of Things and cloud computing to develop the smart health senior care service platform. Senior care service enterprises offer services to the senior through collaboration with the network.

 This study devises the regulatory mechanism for smart senior care services, as depicted in Figure 1. Based on this framework, the study proceeds with the subsequent phase of model construction.

###  Model Assumptions

 This study employs evolutionary game theory to analyze the conflicts of interest and optimal choices among the government, platform, and senior care service enterprises in the context of smart health senior care. The following assumptions and variable settings are established:

 Assumption 1: In the smart health senior care service platform constructed by the government, platform providers, and senior care service enterprises, due to the limited knowledge, time, and energy of the participating parties, they cannot achieve complete rationality in decision-making. Instead, they make optimal or satisfactory decisions with limited information and computing power.^33^ Therefore, this study chooses bounded rationality as the premise for analysis. Game players adopt two behavioral strategies: The government selects between two behavioral strategies: positive regulatory and passive regulation, with probabilities of* x (x*∈*[0*, *1])* and *1-x*, respectively. The platform chooses between two behavioral strategies: opportunism and reciprocity, with probabilities of *y (y*∈*[0, 1])* and *1-y*, respectively. The senior care service enterprises adopt two behavioral strategies: positive cooperation and passive cooperation, with probabilities of *z (z*∈*[0*, *1]) *and *1-z*, respectively. The following will describe the profit and loss under the specific strategies of each entity separately.

 Assumption 2: When the government adopts a positive regulatory strategy, it not only establishes entry thresholds for smart health senior care service platform and senior care service enterprises but also oversees the entire process of construction and operation of smart health senior care service platform. The higher the regulatory efficiency of the government, the lower the likelihood of opportunistic behavior occurring. The regulatory level is *µ*_1_*(µ*_1_∈*[0*,*1])*, assuming that the costs of time and material incurred in the regulatory process are denoted as *C*_g1_, the reputation incentive effect resulting from positive government regulation, and the idealized reward from higher-level government departments are represented by *R*_g1_. Additionally, the feedback on the willingness of senior individuals to use smart senior care services, denoted as *ρ*_1_*(ρ*_1_∈*[0*,*1])*, can be utilized to gauge the status of platform service provision in construction and operation. The actual benefits accrued by the government are *ρ*_1_*R*_g1_. When the government opts for passive regulation, there is no associated cost. However, in cases where violations detrimental to the public interest occur in the operation of the smart health senior care service platform, the government department shall be directly held accountable and sanctioned by the superior department due to inadequate regulation, denoted as *P*_g1_* (P*_g1_*>C*_g1_*)*. Due to the passive impact on society, governance losses, including but not limited to indirect reputation and credibility losses resulting from loss of trust, are denoted as *P*_g2_.

 Assumption 3: The assessment of the construction level of the smart health and senior care service platform determines the capacity for transforming senior care services.^34^ The transformation rate of the platform’s smart aging services is denoted as *ρ*_2 _*(ρ*_2_∈*[0*, *1])*. When the platform chooses a reciprocity strategy to build and operate a smart health senior care service platform, it not only establishes corresponding operational norms for the service platform and implements assessment and management plans for senior care service enterprises within the system, but also allocates manpower, financial resources, and material resources towards infrastructure construction, yielding the basic benefit of *R*_b1_. Concurrently, the highest reward for platform construction and operation by the government is denoted as *R*_b3_, and the government provides subsidies and rewards to the platform based on the construction standards of smart health senior care service platform, calculated as *ρ*_2_*R*_b3_. However, When the platform chooses opportunistic strategies, opportunistic behaviors may arise, such as fraudulent subsidies for platform construction that fail to meet the standard of intelligence, unreasonable fees levied on the senior, and unfair and mutually beneficial cooperation with senior care service enterprises, resulting in the basic benefit of *R*_b2_. The maximum punishment imposed by the government’s positive regulation for the platform is denoted as *P*_b1_. Depending on the effectiveness of government’s positive regulation, the platform incurs the punishment of *µ*_1_*P*_b1_ for opportunistic construction and operation.

 Assumption 4: Based on the capabilities of senior care service enterprises, the age range of senior individuals they can serve is limited, and service age coverage rate of senior care service enterprises is denoted as *ρ*_3_*(ρ*_3_∈*[0*,*1])*. When senior care service enterprises opt to actively cooperate, they collaboratively establish the convenient and cost-effective smart senior care service system with the platform and receive basic benefits, denoted as *R*_h1_. Due to their positive impact on social senior care services through positive cooperation, the maximum reward that senior care service enterprises can receive when actively regulated by government departments is denoted as *R*_h3_. Based on the age coverage of senior care service enterprises, rewards will be given as *ρ*_3_*R*_h3_. Conversely, if senior care service enterprises choose to cooperate passively, they may engage in behaviors such as providing products and services that do not meet the expected value or standards of leaking user information, and obtaining basic benefits denoted as *R*_h2_. Passive cooperation of senior care service enterprises results in low efficiency of the smart health senior care service platform and warrants the highest punishment through positive regulation by the government, denoted as *P*_h1_. Depending on the effectiveness of positive government regulation, the platform will incur the punishment denoted as *µ*_1_*P*_h1_ for opportunistic construction and operation.

 Assumption 5: When the platform opts for reciprocity and senior care service enterprises chooses positive cooperation, both parties gain additional benefits, denoted as* R*_b4 _and *R*_h4_. Based on the performance of both parties, the additional benefits acquired by both parties are denoted as *ρ*_2_*R*_b4 _and *ρ*_3_*R*_h4_.

 Based on the above description and assumptions, a parameter explanation table is provided in Table 1, and a three party strategic game payoff matrix is derived in Table 2.

**Table 1:** Parameter Description

**Symbol**	**Meaning**
*ρ*_1_	Willingness of senior people to use smart senior care services
*R*_g1_	Ideal rewards obtained through positive government regulation
*P*_g1_	The government's passive regulation has been directly held accountable and punished by higher-level departments
*P*_g2_	Indirect losses caused by negative government regulation
*C*_g1_	The cost of positive government regulation
*µ*_1_	The level of positive government regulation
*ρ*_2_	Platform smart aging friendly construction service conversion rate
*R*_b1_	The basic benefits of platform reciprocity construction and operation
*R*_b2_	The basic benefits of opportunistic platform construction and operation
*R*_b3_	The highest reward for platform reciprocity behavior under the background of positive government regulation
*R*_b4_	The platform chooses reciprocity as an additional benefit
*P*_b1_	Punishment for opportunistic behavior of platform under the background of positive government regulation
*ρ*_3_	Platform service provider service coverage
*R*_h1_	The basic benefits of positive cooperation among senior care service enterprises
*R*_h2_	The basic benefits of passive cooperation among senior care service enterprises
*R*_h3_	Under the background of positive government regulation, senior care service enterprises positive cooperation and receive the highest rewards
*R*_h4_	Additional benefits for senior care service enterprises choosing positive cooperation
*P*_h1_	Punishment for passive cooperation among senior care service enterprises under the background of positive government regulation

**Table 2:** Tripartite Game Payment Matrix

**Senior Care Service Enterprises**	**Government Positive Regulation (x)**	
	**Platform**	
	**Reciprocity (y)**	**Opportunism (1-y)**
Positive cooperation (z)	$$\begin{array}{l} \rho_{1}R_{g1}-C_{g1}\\ \rho_{2}R_{b3}+R_{b1}+\rho_{2}R_{b4}\\ \rho_{3}R_{h3}+R_{h1}+\rho_{3}R_{h4} \end{array}$$	$$\begin{array}{l} \rho_{1}R_{g1}-C_{g1}\text{+}\mu_{1}P_{h1}\\ \rho_{2}R_{b3}+R_{b1}\\ -\mu_{1}P_{h1}+(1-\mu_{1})R_{h2} \end{array}$$
Passive cooperation (1-z)	$$\begin{array}{l} \rho_{1}R_{g1}-C_{g1}+\mu_{1}P_{b1}\\ \;-\mu_{1}P_{b1}+(1-\mu_{1})R_{b2}\\ \rho_{3}R_{h3}+R_{h1} \end{array}$$	$$\begin{array}{l} \rho_{1}R_{g1}-C_{g1}+\mu_{1}P_{b1}+\mu_{1}P_{h1}\\ \;-\mu_{1}P_{b1}+(1-\mu_{1})R_{b2}\\ -\mu_{1}P_{h1}+(1-\mu_{1})R_{h2} \end{array}$$
**Senior Care Service Enterprises**	**Government Passive Regulation (1-x)**	
	**Platform**	
	**Reciprocity (y)**	**Opportunism (1-y)**
Positive cooperation (z)	$$\begin{array}{l} 0\\ R_{b1}+\rho_{2}R_{b4}\\ R_{h1}+\rho_{3}R_{h4} \end{array}$$	$$\begin{array}{l} -P_{g1}-P_{g2}\\ R_{b1}\\ R_{h2} \end{array}$$
Passive cooperation (1-z)	$$\begin{array}{l} -P_{g1}-P_{g2}\\ R_{b2}\\ R_{h1} \end{array}$$	$$\begin{array}{l} -P_{g1}-P_{g2}\\ R_{b2}\\ R_{h1} \end{array}$$

###  Model Analysis

 According to the stability theorem of differential equations, two conditions should be satisfied for the stable point of the equation of replication dynamics: *F(x) = 0 *and *dF(x)/dx<0*.

 The expected benefits of positive and passive government regulation (E_11_ and E_12_) are:


(1) $$\left\{\begin{array}{c} E_{\text{11}}=yz(\rho_{1}R_{g1}-C_{g1})+y\left(1-z\right)(\rho_{1}R_{g1}-C_{g1}\text{+}\mu_{1}P_{h1})+\left(1-y\right)z(\rho_{1}R_{g1}-C_{g1}+\mu_{1}P_{b1})+\\ \left(1-y\right)\left(1-z\right)(\rho_{1}R_{g1}-C_{g1}+\mu_{1}P_{b1}+\mu_{1}P_{h1})\\ E_{\text{12}}=\;y\left(1-z\right)\left(-P_{g1}-P_{g2}\right)+\left(1-y\right)z\left(-P_{g1}-P_{g2}\right)+\left(1-y\right)\left(1-z\right)\left(-P_{g1}-P_{g2}\right)\\ \bar{E_{1}}=xE_{\text{11}}+(1-x)E_{\text{12}} \end{array}\right\}$$

Therefore, the evolutionary game replication dynamic equation of government strategy is:


(2) $$\begin{array}{c} F\left(x\right)=dx/dt=x\left(1-x\right)\;\left(E_{11}-\;E_{12}\right)\\ =x(1-x)(-C_{g1}+(P_{b1}(1+y)+P_{h1}(1+z))\mu_{1}+R_{g1}\rho_{1}-(P_{g1}+P_{g2})(yz-1)) \end{array}$$

(3) $$F'\left(x\right)=\left(1-2x\right)(-C_{g1}+(P_{b1}(1+y)+P_{h1}(1+z))\mu_{1}+R_{g1}\rho_{1}-(P_{g1}+P_{g2})(yz-1))$$

Assuming,


$$W\left(y\right)=(-C_{g1}+(P_{b1}(1+y)+P_{h1}(1+z))\mu_{1}+R_{g1}\rho_{1}-(P_{g1}+P_{g2})(yz-1))$$

when $y*=\frac{-C_{g1}+P_{h1}(1+z)\mu_{1}+\rho_{1}R_{g1}+P_{g1}+P_{g2}+P_{b1}\mu_{1}}{\left(P_{g1}+P_{g2}\right)z-P_{b1}\mu_{1}}$ ,

 proposition 1 holds.

 Proposition 1: When 0<y * <y<1, *x* = 1 is the evolutionarily stable point; when 0<y<y * <1,* x* = 0 is the evolutionarily stable point.

 Proof: The function W(y) exhibits a monotonic decrease over the interval. Therefore, when y = y *, *W(y) = 0*, *F(x) = 0*, indicating regardless of the probability of the government choosing positive or passive regulation changes, the government’s strategy will remain unchanged over time. When 0<y * <y<1,* F’(x)|*_x = 0_*>0*,* F’(x)*|_x = 1_*<0*, *x = 1* exhibits stability; when 0<y<y * <1, *F’(x)*|_x = 0_*<0*, *F’(x)*|_x = 1_*>0*, *x = 0* exhibits stability.

 The stability analysis of the platform and senior care service enterprises is consistent with the government analysis method, and the evolutionary game replication dynamic equation can be found in Supplementary file 1.

## Results

###  Stability Analysis of Strategy Combinations

 The Jacobian matrix of the replicated dynamic equation is expressed as Eq. (4) The elements of this matrix are detailed in Supplementary file 2.


(4) $$J=\left[\begin{array}{ccc} F_{x}^{'}(x) & F_{y}^{'}(x) & F_{z}^{'}(x)\\ F_{x}^{'}(y) & F_{y}^{'}(y) & F_{z}^{'}(y)\\ F_{x}^{'}(z) & F_{y}^{'}(z) & F_{z}^{'}(z) \end{array}\right]$$

According to the Lyapunov criterion (indirect method), when all eigenvalues of the Jacobian matrix λ<0, the equilibrium point is asymptotically stable. The characteristic values and stability of each equilibrium point are presented in Table 3.

**Table 3:** Stability Analysis of Trilateral Party Evolutionary Game

**Equilibrium Point**	**Eigenvalue**	**Symbol**	**Stability**
E_1_(0,0,0)	$$\begin{array}{l} -C_{g1}+\rho_{1}R_{g1}+P_{g1}+P_{g2}+P_{b1}\mu_{1}+P_{h1}\mu_{1}\\ R_{b1}-R_{b2}\\ R_{h1}-R_{h2} \end{array}$$	(+,X,X)	Unstable
E_2_(0,0,1)	$$\begin{array}{l} -C_{g1}+\rho_{1}R_{g1}+P_{g1}+P_{g2}+P_{b1}\mu_{1}+2P_{h1}\mu_{1}\\ R_{b1}+\rho_{2}R_{b4}-R_{b2}\\ R_{h2}-R_{h1} \end{array}$$	(+,X,X)	Unstable
E_3_(0,1,0)	$$\begin{array}{l} -C_{g1}+\rho_{1}R_{g1}+P_{g1}+P_{g2}+2P_{b1}\mu_{1}+P_{h1}\mu_{1}\\ -R_{b1}+R_{b2}\\ R_{h1}+\rho_{3}R_{h4}\text{-}R_{h2} \end{array}$$	(+,X,X)	Unstable
E_4_(0,1,1)	$$\begin{array}{l} -C_{g1}+\rho_{1}R_{g1}+2P_{b1}\mu_{1}+2P_{h1}\mu_{1}\\ -R_{b1}-\rho_{2}R_{b4}+R_{b2}\\ -R_{h1}-\rho_{3}R_{h4}+R_{h2} \end{array}$$	(X,X,X)	ESS in condition V
E_5_(1,0,0)	$$\begin{array}{l} C_{g1}-\rho_{1}R_{g1}-P_{g1}-P_{g2}-P_{b1}\mu_{1}-P_{h1}\mu_{1}\\ R_{b1}+\rho_{2}R_{b3}-R_{b2}\text{+}\mu_{1}(R_{b2}\text{+}P_{b1})\\ R_{h1}+\rho_{3}R_{h3}-R_{h2}\text{+}\mu_{1}P_{h1}\text{+}\mu_{1}R_{h2} \end{array}$$	(-,X,X)	ESS in condition I
E_6_(1,0,1)	$$\begin{array}{l} C_{g1}-\rho_{1}R_{g1}-P_{g1}-P_{g2}-P_{b1}\mu_{1}-2P_{h1}\mu_{1}\\ R_{b1}+\rho_{2}\left(R_{b3}+R_{b4}\right)-R_{b2}\text{+}\mu_{1}(R_{b2}\text{+}P_{b1})\\ -R_{h1}-\rho_{3}R_{h3}+R_{h2}-\mu_{1}P_{h1}-\mu_{1}R_{h2} \end{array}$$	(-,X,X)	ESS in condition II
E_7_(1,1,0)	$$\begin{array}{l} C_{g1}-\rho_{1}R_{g1}-P_{g1}-P_{g2}-2P_{b1}\mu_{1}-P_{h1}\mu_{1}\\ -R_{b1}-\rho_{2}R_{b3}+R_{b2}-\mu_{1}(R_{b2}\text{+}P_{b1})\\ R_{h1}+\rho_{3}R_{h3}+\rho_{3}R_{h4}-R_{h2}\text{+}\mu_{1}P_{h1}\text{+}\mu_{1}R_{h2} \end{array}$$	(-,X,X)	ESS in condition III
E_8_(1,1,1)	$$\begin{array}{l} C_{g1}-\rho_{1}R_{g1}-2P_{b1}\mu_{1}-2P_{h1}\mu_{1}\\ -R_{b1}-\rho_{2}\left(R_{b3}+R_{b4}\right)+R_{b2}-\mu_{1}(R_{b2}\text{+}P_{b1})\\ -R_{h1}-\rho_{3}R_{h3}-\rho_{3}R_{h4}+R_{h2}-\mu_{1}P_{h1}-\mu_{1}R_{h2} \end{array}$$	(X,X,X)	ESS in condition IV

Note: X denotes uncertain of symbol. Abbreviation: ESS, Evolutionarily Stable Strategy. Condition I: $R_{b1}+\rho_{2}R_{b3}<R_{b2}-\mu_{1}(R_{b2}+P_{b1}),R_{h1}+\rho_{3}R_{h3}<R_{h2}-\mu_{1}(R_{h2}+P_{h1})$ Condition II: $R_{b1}+\rho_{2}(R_{b3}+R_{b4})<R_{b2}-\mu_{1}\left(R_{b2}\text{+}P_{b1}\right),R_{h1}+\rho_{3}R_{h3}>R_{h2}-\mu_{1}\left(R_{h2}+P_{h1}\right)$ Condition III: $R_{b1}+\rho_{2}R_{b3}>R_{b2}-\mu_{1}\left(R_{b2}+P_{b1}\right),R_{h1}+\rho_{3}(R_{h3}+R_{h4})<R_{h2}-\mu_{1}\left(R_{h2}+P_{h1}\right)$ Condition IV: $\begin{array}{l} C_{g1}<\rho_{1}R_{g1}+2P_{b1}\mu_{1}+2P_{h1}\mu_{1},\\ R_{b1}+\rho_{2}(R_{b3}+R_{b4})>R_{b2}-\mu_{1}\left(R_{b2}\text{+}P_{b1}\right),R_{h1}+\rho_{3}(R_{h3}\text{+}R_{h4})>R_{h2}-\mu_{1}\left(R_{h2}\text{+}P_{h1}\right) \end{array}$ Condition V: $C_{g1}>\rho_{1}R_{g1}+2P_{b1}\mu_{1}+2P_{h1}\mu_{1},R_{b1}\text{+}\rho_{2}R_{b4}>R_{b2},R_{h1}+\rho_{3}R_{h4}>R_{h2}$

 When condition I is met, under the background of positive government regulation, the disparity between the actual basic benefits and actual penalties of opportunism on the platform exceeds the sum of the basic benefits and actual rewards generated by reciprocity, leading the platform to incline towards opportunism. Similarly, the disparity between the actual basic benefits and actual penalties of passive cooperation among senior care service enterprises surpasses the sum of the basic benefits and actual rewards obtained through positive cooperation. Consequently, senior care service enterprises are inclined towards passive cooperation. Therefore, the evolutionarily stable strategy for the three parties is (positive regulation, opportunism, passive cooperation).

 When condition II is met, building upon condition I, if the regulatory level of the government *μ*_1_ enhances, the actual benefits of senior care service enterprises opting for passive cooperation decrease. Alternatively, senior care service enterprises can expand service coverage *ρ*_3_. The actual total benefits of positive cooperation by senior care service enterprises then surpass the disparity between the actual basic benefits of passive cooperation and the actual penalty received. Consequently, when the actual total benefits of platform reciprocity fall short of the disparity between the actual benefits of opportunism and the actual penalty received, the platform tends to opt for opportunism, while senior care service enterprises are inclined towards positive cooperation. Therefore, the evolutionarily stable strategy for the three parties is (positive regulation, opportunism, positive cooperation).

 When condition III is met, building upon condition I, if the regulatory level of the government *μ*_1 _ enhances, the actual benefits of opportunism chosen by the platform are reduced. Alternatively, the platform can increase the conversion rate of smart and aging-friendly construction services *ρ*_2_. When the disparity between the actual basic benefits of opportunism and the actual penalty is less than the sum of the basic benefits and actual rewards generated by reciprocity, the platform tends to choose reciprocity. Furthermore, if the disparity between the actual basic benefits and actual punishments of passive cooperation among senior care service enterprises exceeds the sum of the basic benefits and actual rewards obtained through positive cooperation, senior care service enterprises tend to choose passive cooperation. Therefore, the evolutionarily stable strategy for the three parties is (positive regulation, reciprocity, passive cooperation).

 When condition IV is met, building upon conditions II or III, the government tends to continue positive regulation if the cost of positive regulation is less than the sum of the actual rewards obtained and the punishment income obtained from regulation. Under the context of positive government regulation, if the government increases the rewards for platform reciprocity behavior and positive cooperation with senior care service enterprises, or intensifies the punishment for opportunistic behavior and passive cooperation with senior care service enterprises, the actual total benefits of platform reciprocity will surpass the difference between the actual benefits of opportunism and the actual punishment received. Moreover, if the actual total benefits of positive cooperation with senior care service enterprises outweigh the difference between the actual basic benefits of passive cooperation and the actual punishment received, platform tends to choose reciprocity, while senior care service enterprises tend to opt for positive cooperation. Therefore, the evolutionarily stable strategy for the three parties is (positive regulation, reciprocity, positive cooperation).

 When condition V is met, building upon condition IV, if the actual total benefit of reciprocity chosen by the platform when cooperating with senior care service enterprises exceeds the basic benefit of opportunism, and the actual total benefit of positive cooperation chosen by senior care service enterprises surpasses the basic benefit of passive cooperation, then the platform party will tend to choose reciprocity, and senior care service enterprises tend to opt for positive cooperation. As the platform and senior care service enterprises continue to opt for positive cooperation, the government can achieve certain benefits. However, when the cost of positive government regulation exceeds the sum of the actual rewards obtained and the punishment income obtained from regulation, the government tends to shift towards passive regulation. Therefore, the evolutionarily stable strategy for the three parties is (passive regulation, reciprocity, positive cooperation).

###  Initial Value Assignment of Parameters

 To set the simulation model parameters reasonably, based on the above analysis results and referring to relevant research, news examples, and data pertaining to the construction of smart health senior care service platform. The initial values to the parameters are assigned accordingly.

 The development of smart health senior care facilities in China is in its developmental phase, selecting data related to smart health senior care in China can better demonstrate the evolution process of game party strategies. Firstly, the parameters are set based on literature and actual surveys. According to the survey by the National Senior Network, the total retirement pension of the senior exceeds 8 trillion yuan, and the consumption demand in the senior healthcare industry market is over 5 trillion yuan. Therefore, the willingness to use smart senior care services for the senior is set as *ρ*_1_ = 0.6; According to incomplete statistics from Agedata, as of the end of 2023, investment in the silver hair industry is accelerating its diversified layout, and the senior care technology and smart senior care service industry are far ahead, accounting for 33.5%.^35^ Therefore, the conversion rate of platform smart aging services is set as *ρ*_2_ = 0.3; From 2011 to 2023, 33 policies related to smart senior care were introduced at the national level, of which 6 explicitly mentioned the regulation of senior care service institutions. Therefore, the level of positive government regulation is set as *μ*_1_ = 0.2. According to Li et al,^36^ it can be seen that the overall development level of the senior care industry in 41 cities in the Yangtze River Delta region has increased to the comprehensive score of 0.323, the coverage rate of senior care service provider services is set as *ρ*_3_ = 0.3, referring to the disclosure data and related research of urban smart senior care services in the Yangtze River Delta region in China,^37^ the penalty of direct accountability from higher authorities for passive government regulationis set as* P*_g1_ = 40, indirect losses caused by passive government regulation is set as *P*_g2_ = 20.

 Secondly, assuming *P*_g1 _> *C*_g1_ in the previous analysis, the cost of government positive regulation is set as* C*_g1_ = 25, and the initial benefits of government regulation on the platform are less than the cost, that is, the ideal reward for government positive regulation is set as *R*_g1_ = 40. According to the stability condition I of E_5 _(1, 0, 0), set the initial parameters. According to *R*_b1 _*+ ρ*_2_*R*_b3_*<R*_b2_*-μ*_1_*(R*_b2 _*+ P*_b1_*)*, the basic benefit of platform reciprocity construction and operation is set as *R*_b1_ = 18, and the basic benefit of platform opportunism construction and operation is set as *R*_b2_ = 48. The highest reward for platform reciprocity behavior under the background of positive government regulation is *R*_b3_ = 5, and the punishment for platform opportunism behavior under the background of positive government regulation is *P*_b1_ = 5. According to *R*_h1 _*+ ρ*_3_*R*_h3_*<R*_h2_*-μ*_1 _*(R*_h2 _*+ P*_h1_*)*, the basic benefit of positive cooperation between senior care service enterprises is set as *R*_h1_ = 18, and the basic benefit of passive cooperation between senior care service enterprises is set as *R*_h2_ = 42. Under the background of positive government regulation, senior care service enterprises receive the highest reward for positive cooperation is set as *R*_h3_ = 5. Under the background of positive government regulation, the punishment received by senior care service enterprises for passive cooperation is set as *P*_h1_ = 5. And because the initial state of the platform chooses opportunism and senior care service enterprises chooses passive cooperation, additional benefits for both parties *R*_b4 _and *R*_h4_ is set as *R*_b4_ = *R*_h4_ = 0. Due to the combination of real data and simulation values used in this study to validate and discuss the model, there may be deviations in the model analysis. When the validation values exceed the range of simulation values, there may be scenarios where the model predictions are not valid. The following is the numerical simulation of the evolutionary trajectories of each game player using MATLABR2021a, the code (partial) can be found in Supplementary file 3.

###  Model Validation

**Figure 2:**
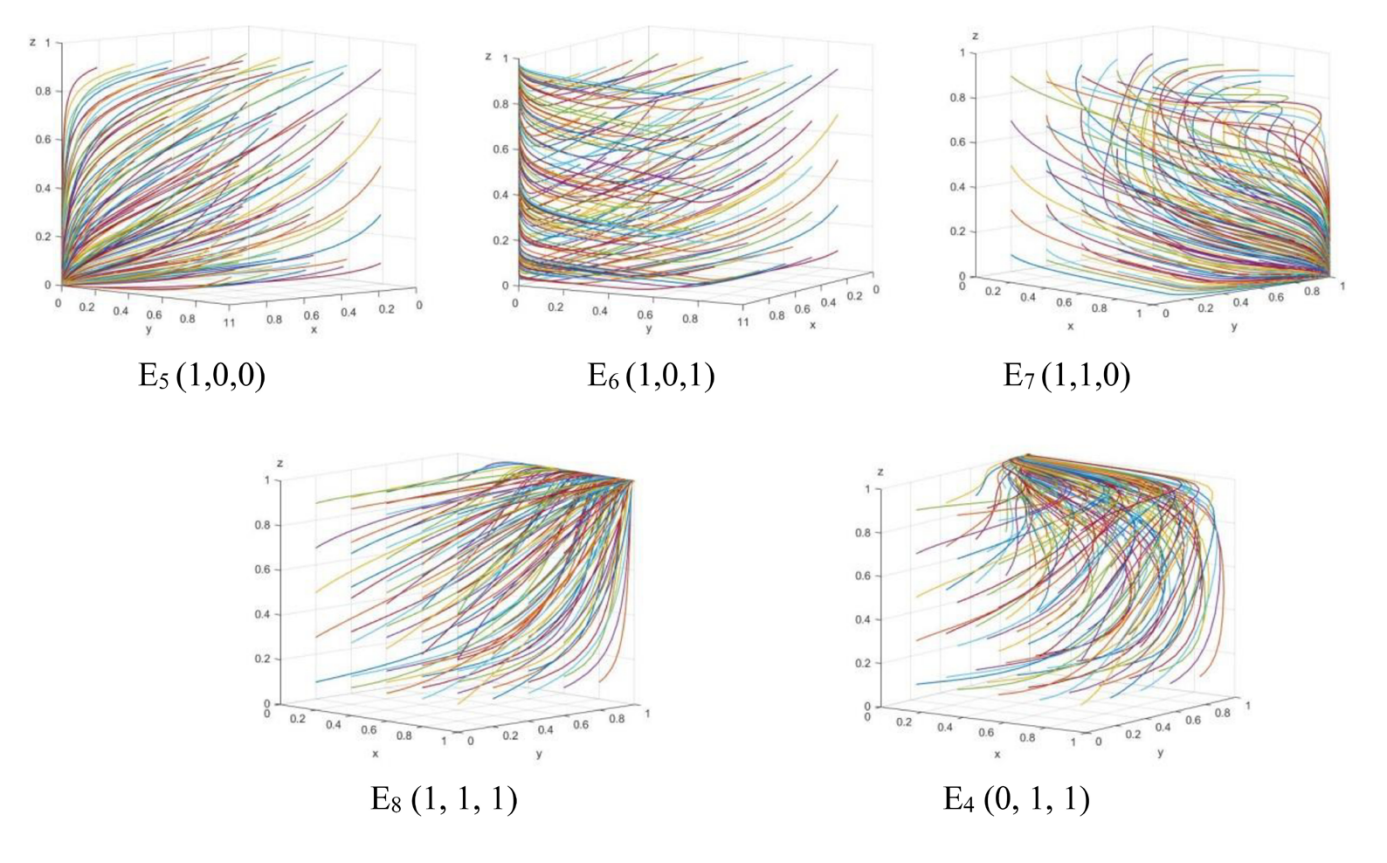
Evolutionary Equilibrium Diagram. On the graph, the x-axis, y-axis, and z-axis represent the probabilities of government, platform, and senior care service enterprises selection strategies, respectively.

 When condition I is met, and the parameters values match the initial value assignment, the strategy evolution is shown in Figure 2. In the early stages of establishing the smart health senior care service platform, the platform and senior care service enterprises did not have the clear long-term understanding of the platform’s operation. To maintain basic interests, the platform owner remained stable in opportunistic strategies. When the platform chooses opportunism, driven by interests, senior care service enterprises will remain stable in the passive cooperation strategy. At this time, the government will increase regulation of the platform and senior care service enterprises to promote the development of smart health senior care service platform. So the system strategy will stabilize at E_5_ (1, 0, 0).

 When condition II is met, with parameters values adjusted as follows: *R*_b1_ = 20, *R*_b2_ = 45, *R*_b3_ = 10, *μ*_1_ = 0.3, *P*_b1_ = 20, *ρ*_3_ = 0.6, *R*_h3_ = 10, *P*_h1_ = 20, and others are the same as condition I. The strategy evolution is shown in Figure 2. It can be observed that in the early stages of establishing the smart health senior care service platform, the platform will gradually stabilize in the opportunistic strategy because the benefits obtained from reciprocity are smaller than the actual benefits obtained from opportunism. Meanwhile, senior care service enterprises increase service coverage during positive government regulation *ρ*_3_. When the basic benefits and subsidies obtained from positive cooperation outweigh the benefits from passive cooperation, senior care service enterprises will remain stable in the positive cooperation strategy. At this time, the government will maintain stability in the positive regulatory strategy due to unstable platform construction and operation. Consequently, the system strategy will stabilize at E_6_ (1, 0, 1).

 When condition III is met, with parameters values adjusted as follows: *R*_b1_ = 30, *ρ*_2_ = 0.6, *R*_b2_ = 45, *R*_b3_ = 20, *μ*_1_ = 0.3,* P*_b1_ = 20, *R*_h1_ = 15, *ρ*_3_ = 0.2, *R*_h3_ = 20, *R*_h2_ = 40, *P*_h1_ = 20, and others are the same as condition I. The strategy evolution is shown in Figure 2. It can be observed that the platform increases the service conversion rate under positive government regulation *ρ*_2_. The actual benefits obtained by reciprocity outweigh the benefits sought by opportunism, and the platform side will remain stable in the reciprocity strategy. At this time, the government will maintain stability in the positive regulatory strategy due to unstable platform construction and operation. Consequently, the system strategy will stabilize at E_7 _(1, 1, 0).

 When condition IV is met, with parameters values adjusted as follows: *ρ*_2_ = 0.6,* R*_b2_ = 45, *R*_b4_ = 10, *μ*_1_ = 0.5, *ρ*_3_ = 0.6, *R*_h4_ = 10, *R*_h2_ = 42, and others are the same as condition I. The strategy evolution is shown in Figure 2. With the transformation rate of smart and aging-friendly construction services *ρ*_3 _increasing, the actual benefits obtained by platform reciprocity are greater than those obtained by opportunism, and the platform will remain stable in choosing reciprocity strategies. At this time, the additional benefits of both the platform and senior care service enterprises choosing the positive strategy increase, so senior care service enterprises will also remain stable and actively cooperate. Consequently, the system strategy will stabilize at E_8_ (1, 1, 1).

 When condition V is met, with parameters values adjusted as follows:* C*_g1_ = 30, *R*_g1_ = 40, *R*_b1_ = 50, *R*_b3_ = 10, *R*_b4_ = 10, *μ*_1_ = 0.3, *P*_b1_ = 10, *R*_h1_ = 50, *R*_h3_ = 10, *R*_h4_ = 10, *R*_h2_ = 45, *P*_h1_ = 10, and others are the same as condition IV. The strategy evolution is shown in Figure 2. It can be observed that in the mature stage of the development of smart health senior care service platform, the cost of government regulation is high and the benefits obtained reach the limit. As the platform and senior care service enterprises increase their awareness of smart senior care, as well as the benefits of mutual reciprocity and positive cooperation, the platform and senior care service enterprises can also choose the win-win strategy when the government does not regulate. Consequently, the system strategy will stabilize at E_4_ (0, 1, 1).

## Discussion

 The analysis examines the impact of significant parameter variations on the strategic choices of game participants.

###  The Impact of Government’s Positive Regulation Level

**Figure 3:**
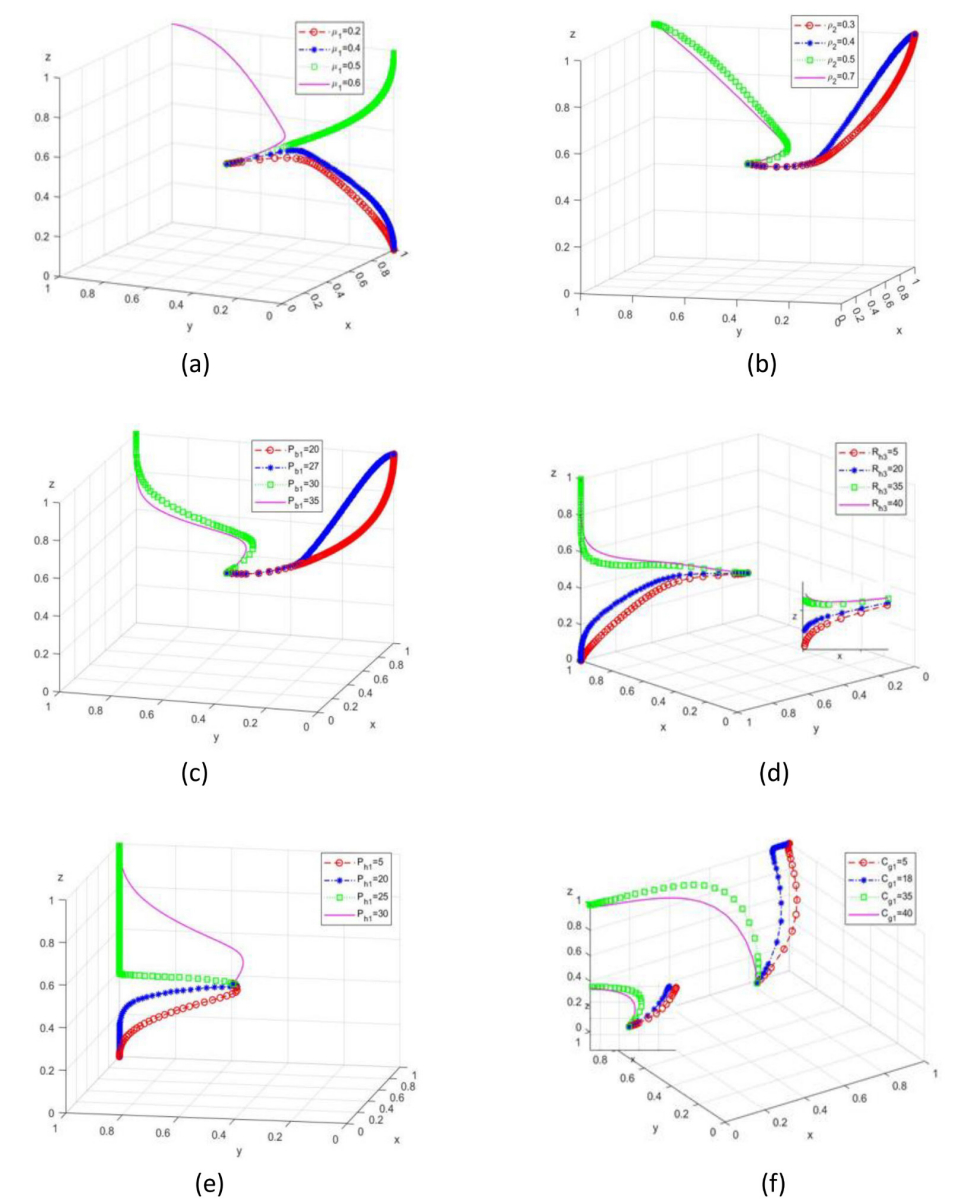
Parameter Sensitivity Analysis.

 Based on condition I, let *μ*_1_ = {0.2, 0.4, 0.5, 0.6}, the result is shown in Figure 3a. There is the positive correlation between *μ*_1_ and the probability of senior care service enterprises choosing positive cooperation, and the critical value for senior care service enterprises to change their strategies is *μ*_1_ = 0.48. There is the positive correlation between *μ*_1_ and the probability of the platform choosing reciprocity. The platform shifts from opportunism to reciprocity, and the critical value for the platform to change its strategy is *μ*_1_ = 0.54. Due to changes in the strategies of the platform and service providers, the evolution strategy of the entire system may also change. The system’s strategy may shift from (positive regulation, opportunism, passive cooperation) to (positive regulation, opportunism, positive cooperation), and ultimately stabilize at (positive regulation, reciprocity, positive cooperation).When *μ*_1 _< 0.48, as *μ*_1 _increases, the speed at which senior care service enterprises stabilize and actively cooperate increases, and the speed at which the government stabilizes and actively regulates also increases. When 0.48 < *μ*_1 _< 0.54, as *μ*_1 _increases, the speed at which the platform stabilizes towards reciprocity increases, and senior care service enterprises choose to actively cooperate. When *μ*_1 _> 0.54, as *μ*_1_ increases, the speed at which the platform side stabilizes towards reciprocity and senior care service enterprises stabilizes towards positive cooperation decreases, and the speed at which the government stabilizes towards positive regulation also decreases. This indicates that the level of government regulation should be appropriately improved to make the platform side tend towards reciprocity and senior care service enterprises tend towards positive cooperation, thereby achieving the stable state of the system’s positive strategy selection between the platform side and senior care service enterprises under positive government regulation.

###  Impact of Platform Smart Aging Adaptation Construction Service Conversion Rate

 Based on condition II, let *ρ*_2_ = {0.3, 0.4, 0.5, 0.7}, the result is shown in Figure 3b. *ρ*_2_ is positively correlated with the probability of platform choosing reciprocity. The critical value for the platform to shift from opportunism to reciprocity is *ρ*_2_ = 0.45. Due to the strategy change of the platform, the evolution strategy of the entire system may also change, and the system’s strategy may shift from (positive regulation, opportunism, positive cooperation) to (positive regulation, reciprocity, positive cooperation). When *ρ*_2 _< 0.45, with the increase of *ρ*_2_, the speed of the platform stabilized at reciprocity increases. When *ρ*_2 _> 0.45, with the increase of *ρ*_2_, the speed at which the platform stabilizes reciprocity decreases. It shows that under the background of positive government regulation, if the platform increases the conversion rate of smart and age-appropriate construction services, the platform side will tend to reciprocity, and achieve the stable state of positive government regulation and reciprocal development of the platform side.

###  Impact of Opportunistic Punishment on the Platform

 Based on condition II, let *P*_b1_ = {20, 27, 30, 35}, the results are shown in Figure 3c. *P*_b1 _is positively correlated with the probability of the platform choosing reciprocity. The critical value for the platform to shift from opportunism to reciprocity is* P*_b1_ = 29. As the platform’s strategy changes, the system’s strategy may shift from (positive regulation, opportunism, positive cooperation) to (positive regulation, reciprocity, positive cooperation). When *P*_b1 _< 29, the evolution speed of the platform increases with the increase of *P*_b1_; when *P*_b1 _> 29, the evolution speed of the platform decreases with the increase of *P*_b1_. It is suggested that the punishment for the opportunism of the platform should be properly increased, which can promote the platform to tend to reciprocity, and achieve the stable state of positive regulation by the government and reciprocal development of the platform.

###  The Impact of Passive Cooperation Penalties on Senior Care Service Enterprises

 Based on condition III, let *P*_h1_ = {5, 20, 25, 30}, the results are shown in Figure 3d. *P*_h1_ is positively correlated with the probability of senior care service enterprises choosing positive cooperation. The critical value for senior care service enterprises to shift their strategy from passive cooperation to positive cooperation is *P*_h1_ = 23. The systematic strategy may shift from (positive regulation, reciprocity, passive cooperation) to (positive regulation, reciprocity, positive cooperation). When *P*_h1_ = 23, senior care service enterprises evolves to the stable speed with the increase of *P*_h1_; when *P*_h1_>23, the senior care service enterprises evolves to the stable speed with the increase of *P*_h1_. It indicates that the punishment for passive cooperation of senior care service enterprises should be appropriately increased, which can promote senior care service enterprises to choose positive cooperation and achieve the stable state of positive government regulation and positive cooperation of senior care service enterprises.

###  The Impact of Rewards for Positive Cooperation Among Senior Care Service Enterprises

 Based on condition III, let *R*_h3_ = {5, 20, 35, 40}, the results are shown in Figure 3e. *R*_h3_ is positively correlated with the probability of senior care service enterprises choosing positive cooperation. The critical value for senior care service enterprises to shift their strategy from passive cooperation to positive cooperation is *R*_h3_ = 25. As senior care service enterprises’ strategy changes, the system’s strategy may shift from (positive regulation, reciprocity, passive cooperation) to (positive regulation, reciprocity, positive cooperation). When *R*_h3_< 25, as *R*_h3 _increase, senior care service enterprises evolve to a stable rate of increase; when *R*_h3_> 25, as *R*_h3 _increase, senior care service enterprises evolve to a stable rate of decreases. It indicates that the rewards for positive cooperation of senior care service enterprises should be appropriately increased, which can promote senior care service enterprises to choose positive cooperation and achieve the stable state of active government regulation and positive cooperation of senior care service enterprises.

###  The Impact of Active Government Regulation on Costs

 Based on condition V, let *C*_g1_ = {5, 18, 35, 40}, the results are shown in Figure 3f. *C*_g1_ is negatively correlated with the probability of government choosing regulation. The critical value for the government to transition from active regulation to passive regulation is *C*_g1_ = 20. As the result of changes in the government’s strategy, the systematic strategy may change from (positive regulation, reciprocity, positive cooperation) to (passive regulation, reciprocity, positive cooperation). When *C*_g1_< 20, as *C*_g1 _increase, the speed of government evolution to stability decreases. When* C*_g1_> 20, as *C*_g1 _increase, the government evolves at the steady rate. When the system is in the state (passive regulation, reciprocity, positive cooperation), the government should properly control the regulatory cost.

## Conclusion

 This study explores the game theory logic relationship of the smart health senior care service platform through model simulation, and explores the optimal stability strategy of the participating parties. The research results indicate that:

 (1) In the process of strategy evolution, while ensuring the existence and development of the platform and senior care service enterprises, the both sides reached cooperation under the background of active regulation by the government, which is the key to the smooth operation of the smart health senior care service platform. Additionally, this study indicates that for the game players to achieve the ideal state (active regulation, reciprocity, positive cooperation), the cost of government regulation should be less than the actual reward of regulation.

 (2) The level of active government regulation is an important factor that affects the selection strategy of platform and senior care service enterprises. Appropriately improving the level of active government regulation is conducive to reducing the speculative behavior of platform and senior care service enterprises. The conversion rate of smart aging construction services and punishment for platform opportunism are important factors affecting the platform’s strategy selection. Encouraging platform technology to improve and increasing the punishment for platform opportunism will increase the probability of the platform choosing reciprocity. This is similar to the conclusion of Ferlie et al^38^ in encouraging companies involved in smart senior care services to develop new technologies. In addition, this study finds that the service coverage of senior care service enterprises, the reward for positive cooperation, and the punishment for passive cooperation are important factors affecting the strategy selection of senior care service enterprises. The reward for positive cooperation of senior care service enterprises increases, the service coverage of senior care service enterprises increases, and the probability of positive cooperation of senior care service enterprises increases, gradually stabilizing to positive cooperation.

 (3) For the platform and senior care service enterprises, regardless of which side bears more costs or gains more actual benefits in the construction and operation of the smart health senior care service platform, government intervention is ultimately necessary to guide the system towards the ideal state E_8_(1, 1, 1). This aligns with the policy recommendations proposed by Côté-Boileau,^39^ suggesting that the development of smart health senior care services requires robust policies and systems. Active government regulation can incentivize the platform and senior care service enterprises to actively engage in platform construction. Simultaneously, the quality of the smart health senior care service platform is improved, which corroborates the findings of Secundo et al.^40^ This suggests that the perceived usefulness of the senior will be enhanced, leading to increased willingness to use such services. Consequently, the government stands to benefit, prompting further improvement in regulatory levels, thus forming the closed loop of continuous improvement and development.

 According to the aforementioned conclusions, the recommendations are proposed for the various stakeholders involved in the smart health senior care service platform:

 (1) The government should assume the guiding role. By establishing a regulatory framework and imposing severe penalties for illegal or irregular behavior, the likelihood of opportunistic behavior among participants can be reduced. Furthermore, the government should incentivize active participation from the platform and senior care service enterprises, potentially through increased rewards. Clear planning of the development trajectory of smart senior care is essential, guiding participants to recognize the promising development prospects within this sector.

 (2) Enhance the feasibility and motivation for self-assessment and mutual oversight among participants. Drawing from related research on senior regulation^41^ and suggestions for the implementation of a smart electronic procurement system for platform to engage senior care service enterprises,^42^ it is advisable to implement mechanisms for mutual oversight and self-assessment between the platform and senior care service enterprises throughout the platform’s establishment and operation. Such measures are instrumental in fostering the self-regulation advancement of the smart health senior care industry.

 While this study underscores the pivotal role of the government in establishing the smart health senior care service platform and the effectiveness of pertinent regulatory measures, there might still be biases in the behavioral analysis of participants within the platform. In the future, employing a diverse array of methods, coupled with real-world case studies based on multi-source data, could offer further insights. For instance, analyzing game scenarios among similar types of smart senior care service enterprises or exploring partner selection dynamics between platform and senior care service enterprises within the digital landscape could yield valuable insights.

## Ethical issues

 None. This study uses publicly available secondary data.

## Conflicts of interest

 Authors declare that they have no conflicts of interest.

## Supplementary files


Supplementary file 1. Replication Dynamic Equation.

Supplementary file 2. Jacobian Matrix of Replicated Dynamical Systems.

Supplementary file 3. Code (Partial).
