# Supplementary material for: The Impact Mechanism of Government Regulation on the Operation of Smart Health Senior Care Service Platform: A Perspective From Evolutionary Game Theory
Source: Int J Health Policy Manag. 2025 Feb 22;14:8646. doi: 10.34172/ijhpm.8646 (PMC12032266; doi:10.34172/ijhpm.8646)
Supplement: Supplementary file 1 — Replication Dynamic Equation. [file ijhpm-14-8646-s001.pdf]

Article title: The Impact Mechanism of Government Regulation on the Operation of Smart Health Senior Care Service Platform: A Perspective From Evolutionary Game Theory

Journal name: International Journal of Health Policy and Management (IJHPM)

Authors' information: Meng Xiao*, Huan Liu

School of Management, Shenyang University of Technology, Shenyang, China.

***Correspondence to:** Meng Xiao; Email: mengxiao@sut.edu.cn

Citation: Xiao M, Liu H. The impact mechanism of government regulation on the operation of smart health senior care service platform: a perspective from evolutionary game theory. Int J Health Policy Manag. 2025;14:8646. doi:10.34172/ijhpm.8646

Supplementary file 2. Jacobian Matrix of Replicated Dynamical Systems

Jacobian matrix of replicated dynamical systems:

$$J=\begin{bmatrix} \begin{array}{l}(1-2x)(-C_{g1}+(P_{b1}(1+y)+P_{h1}(1+z))\mu_1\\ +R_{g1}\rho_1-(P_{g1}+P_{g2})(yz-1))\end{array} & x(1-x)((P_{b1}\mu_1-(P_{g1}+P_{g2})z) & x(1-x)(P_{h1}\mu_1-(P_{g1}+P_{g2})y) \\ y(1-y)(\rho_2 R_{b3}+\mu_1(R_{b2}+P_{b1})) & \begin{array}{l}(1-2y)(R_{b1}+\rho_2(xR_{b3}+zR_{b4})\\ -R_{b2}+\mu_1 x(R_{b2}+P_{b1}))\end{array} & y(1-y)\rho_2 R_{b4} \\ (1-z)\,z(\rho_3 R_{h3}+\mu_1 P_{h1}+\mu_1 R_{h2}) & (1-z)\,z\rho_3 R_{h4} & \begin{array}{l}(1-2z)(R_{h1}+\rho_3 xR_{h3}+\rho_3 yR_{h4}\\ -R_{h2}+x\mu_1 P_{h1}+\mu_1 R_{h2}x)\end{array} \end{bmatrix}$$
