# Supplementary material for: The Impact Mechanism of Government Regulation on the Operation of Smart Health Senior Care Service Platform: A Perspective From Evolutionary Game Theory
Source: Int J Health Policy Manag. 2025 Feb 22;14:8646. doi: 10.34172/ijhpm.8646 (PMC12032266; doi:10.34172/ijhpm.8646)
Supplement: Supplementary file 2 — Jacobian Matrix of Replicated Dynamical Systems. [file ijhpm-14-8646-s002.pdf]

**Article title:** The Impact Mechanism of Government Regulation on the Operation of Smart Health Senior Care Service Platform: A Perspective From Evolutionary Game Theory

**Journal name:** International Journal of Health Policy and Management (IJHPM)

**Authors' information:** Meng Xiao*, Huan Liu

School of Management, Shenyang University of Technology, Shenyang, China.

***Correspondence to:** Meng Xiao; Email: mengxiao@sut.edu.cn

**Citation:** Xiao M, Liu H. The impact mechanism of government regulation on the operation of smart health senior care service platform: a perspective from evolutionary game theory. Int J Health Policy Manag. 2025;14:8646. doi:10.34172/ijhpm.8646

**Supplementary file 3.** Code (Partial)

Smart senior care service platform regulatory game analysis code (excerpt):

```
function
dydt=yanglao(t,y,rho1,Rg1,Pg1,Pg2,Cg1,mu1,rho2,Rb1,Rb2,Rb4,Rb3,Pb1,rho3,Rh1,Rh2,Rh3,P
h1,Rh4)
dydt=zeros(3,1);
dydt(1)=y(1)*(1-y(1))*((rho1*Rg1-Cg1)-(Pg1+Pg2)*(y(2)*y(3)-1)+ Pb1*mu1 + Ph1*mu1 -
Pb1*mu1*y(2) - Ph1*mu1*y(3) - Pg1*y(2)*y(3) - Pg2*y(2)*y(3));
dydt(2)=y(2)*(1-y(2))*(Rb1+rho2*y(1)*(Rb3+y(3)*Rb4)-Rb2+mu1*y(1)*(Rb2+Pb1));
dydt(3)=y(3)*(1-y(3))*(Rh1+rho3*y(1)*Rh3+rho3*y(1)*Rh4-Rh2+y(1)*mu1*Ph1+mu1*Rh2*y
(1));
end
clear;
Cg1=25;rho1=0.6;Rg1=40;Pg1=40;Pg2=20;mu1=0.3;
Rb1=20;rho2=0.3;Rb2=45;Rb3=10;Rb4=0;Pb1=20;
Rh1=18;rho3=0.6;Rh2=40;Rh3=10;Rh4=0;Ph1=20;
for i =0.1:0.2:1
    for j =0.1:0.2:1
        for k =0.1:0.2:1
        [t,y]=ode45(@(t,y)
yanglao(t,y,rho1,Rg1,Pg1,Pg2,Cg1,mu1,rho2,Rb1,Rb2,Rb3,Rb4,Pb1,rho3,Rh1,Rh2,Rh3,Ph1,Rh
4),[0 50] ,[i j k]);
        figure(2)
        grid on
        plot3(y(:,1),y(:,2),y(:,3),'linewidth',1);
        set(gca,'XTick',[0:0.2:1],'YTick',[0:0.2:1],'ZTick',[0:0.2:1])
```

```
            hold on
            axis([0 1 0 1 0 1])
            view([45 10])
            end
      end
end
xlabel('x','Rotation',0);
ylabel('y','Rotation',0);
zlabel('z','Rotation',360,'position',[0 0 1.05]);
```
